# Validation of the hyperbolic temperament questionnaire in Iran

**DOI:** 10.1186/s40359-023-01364-3

**Published:** 2023-10-05

**Authors:** Saeid Komasi, Zahra Chamandoost, Anis Vaysi, Mohadese Amirian, Hadis Rezaeean, Christopher J. Hopwood

**Affiliations:** 1 Department of Neuroscience and Psychopathology Research, Mind GPS Institute, Kermanshah, Iran; 2 Department of Psychology, University of Zurich, Zürich, Switzerland

**Keywords:** Borderline, Hyperbolic temperament, Personality disorder, Reliability, Validity

## Abstract

**Objective:**

Because of the importance of the cross-cultural study of hyperbolic temperament in increasing knowledge related to borderline personality disorder (BPD), the present study was conducted to test the reliability, construct, criterion, and discriminant validity of the Hyperbolic Temperament Questionnaire (HTQ) in three Iranian samples.

**Methods:**

Using a cross-sectional design, the HTQ 11-item version translated into Farsi was provided to three selected samples (total *N* = 558, 72% female, 18 to 77 years old with an average of 30.2 and a standard deviation of 10.3). The samples included non-personality disorder samples (n = 194), samples with BPD symptoms (n = 104), and samples with other personality disorder symptoms (n = 260). Data were collected using multiple validating measurements. Factor analysis was used to verify that the HTQ is unidimensional and correlations and regression models were used to examine its associations with other constructs.

**Results:**

Factor analysis confirmed the single-factor structure of the HTQ in two non-personality disorder and BPD samples. The internal consistency of all items and the total scale were acceptable across the samples (*α* = 0.87 to 0.91). Positive correlations with maladaptive constructs such as negative affectivity and interpersonal sensitivity and negative correlations with adaptive constructs supported the criterion validity of HTQ. The HTQ was specifically related to borderline symptoms, even after controlling for similar constructs such as depression.

**Conclusion:**

The 11-item version of HTQ has acceptable reliability and validity in Iranian samples. Using this short tool for rapid screening of cases with BPD before common procedures such as clinical interviews helps to save diagnostic time and costs.

**Supplementary Information:**

The online version contains supplementary material available at 10.1186/s40359-023-01364-3.

## Introduction

Hyperbolic temperament refers to an essentially genetic predisposition that contributes significantly to a general tendency to experience and seek validation for negative emotions [1]. Individuals with a hyperbolic temperament try to manage the persistent feeling of inner pain caused by seeking validation from others and tend to take offense when others do not notice their pain [1–3]. Hyperbolic temperament was originally conceived as the underlying genetic predisposition of borderline personality disorder (BPD) literature [2, 4]. In this model, people with hyperbolic temperament transform unbearable feelings of sadness, anger, shame, and fear into relentless efforts to draw the attention of others to the significance of their inner emotional pain [2]. This pattern emerges via kindling events (a range of normative experiences such as the first intimate relationship with someone and traumatic experiences such as childhood and adolescent maltreatment) that heighten emotional arousal and the need for social support [1, 5]. However, this behavior becomes maladaptive because it turns others away and thus fails to effectively regulate emotions [5]. Hyperbolic temperament is strongly associated with the negative affectivity/neuroticism trait [6, 7] and the concept of emotional dysregulation in Linehan’s bio-social theory in patients with BPD [8, 9].

The negative consequences of hyperbolic temperament can vary from normal interpersonal situations to traumatic events [10]. The key feature of hyperbolic temperament is the individual’s interpersonal sensitivity to kindling events, and their difficulties managing emotions that result when interpersonal situations do not go well [5, 7, 11]. Although higher levels of hyperbolic temperament and more toxic kindling events such as childhood maltreatment antecede more severe symptoms of BPD, the persistence of more or fewer symptoms can be influenced by emotional and cognitive symptoms of a temperamental origin and how the interpersonal environment is managed [6, 10]. Thus, the model assumes that BPD symptoms emerge from a transaction between hyperbolic temperament and kindling events in the interpersonal environment [10]. In other words, acute symptomatology in BPD can be the result of a transaction between one of the core characteristics of the disorder such as hyperbolic temperament, and inefficient strategies to modulate this temperament in the face of environmental triggers [12].

Hopwood et al. (2012) developed a questionnaire to measure hyperbolic temperament, with the specific goal of distinguishing this phenomenon from general measures of negative affect such as depression in non-clinical, clinical, and treatment samples from the U.S. [10]. Although items from the original 48-item scale could be described by five factors, including hyperbolic, agentic, passive, validation seeking, and detached components, the first and most central hyperbolic temperament factor had a strong significant relationship with the neuroticism and emotional neglect and abuse [10]. This factor consists of eleven items and includes the experience of negative moods and hyperbolic responses to these moods [5]. Although this measure functions well in U.S. samples, very little is known about its properties in different societies and cultures [7, 10]. To our knowledge, no research in other cultures has tested the reliability and validity of the questionnaire in non-U.S. samples. Thus, the goal of this study was to examine the psychometric properties of the Hyperbolic Temperament Questionnaire (HTQ) in an Iranian population. Specifically, we first aimed to test the construct validity of the questionnaire using factor analysis methods. The second objective was to test the reliability of the items and the total scale using Cronbach’s alpha. The third aim was to test criterion validity (correlation with other valid questionnaires) and discriminant validity (distinguishing the sample with borderline personality symptoms from the non-clinical group).

## Methods

### Translation process

Our first step was to translate the 11-item HTQ into Farsi. After contacting and obtaining permission from the authors of the original questionnaire, the items were translated into Farsi by a member of the present research team (*SK*). In the next stage, the items were translated back into the original language (i.e., English) by a Ph.D. student in the field of English translation with an IELTS score of 7.5. Then, the validity of the translation was confirmed in interaction with the first author of the original questionnaire (*CH*). When the items were translated from Farsi to English, the wording of the six questions was slightly different from the original version. However, discussions between the first authors of the present study and the initial validation study led to assurance of the accuracy of the translation and agreement between them.

### Samples and data collection

We sampled participants from the general population of Kermanshah province in western Iran between May and December 2022. The samples were invited to participate in the study through public calls and non-random and convenience methods. Nearly 700 adults 18 years and older agreed to participate in the study and received the research questionnaires. All these people were free from psychiatric medication and psychotherapy in the last 4 weeks and none of them were addicted to illegal drugs. Overall, 610 questionnaires were returned to the research team. However, 52 subjects were excluded due to missing data on more than 40% of multiple questionnaire items or invalid answers, leaving a final sample of 558. Questionnaires were delivered by four members (*ZC, AV, MA, HR*) of the research team to all participants and all of them returned the completed forms to the research team within 48 h. The age ranged from 18 to 77 years (30.2 ± 10.3) and most of the participants were female (n = 402, 72%), single (n = 307, 55%), had a bachelor’s degree or higher (n = 390, 70%), and were employed or in college (n = 267, 48%). More than one-fifth (21%) of participants reported a history of previous psychological diagnoses including major depressive disorder (4.1%), anxiety disorder (3.6%), obsessive-compulsive disorder (2.5%), panic disorder (0.7%), sleep disorder (3.6%), eating disorder (0.7%), pathological gambling (1.1%), other conditions (1.1%), and multiple concurrent diagnoses (3.6%).

We used recommended cut points to identify cases with significant symptoms of BPD and any other symptoms of the personality disorder according to the fourth version of the Personality Diagnostic Questionnaire (PDQ-4) [13]. Three groups were identified according to the cut-off scores: (i) non-personality disorder samples (NPD; a score below the cut-off points for each personality disorder and a total score ≤ 50 on the PDQ), (ii) samples with borderline personality disorder symptoms (BPD; a score ≥ 5 on the BPD subscale of PDQ regardless of the overall score), and (iii) samples with other personality disorders symptoms (OPD; a score greater than the cut-off points for at least one personality disorder on the PDQ except for BPD along with a score > 20 on the total scale). The characteristics of the three samples and their mean scores in the total PDQ and BPD subscale are as follows: NPDs (n = 194, 79% female, 31 years mean age, mean scores of 14.2 and 0.91 in the PDQ and BPD), BPDs (n = 104, 73% female, 29 years mean age, mean scores of 45.2 and 5.9 in the PDQ and BPD), OPDs (n = 260, 66% female, 30 years mean age, mean scores of 35.2 and 2.6 in the PDQ and BPD). By identifying three groups using the PDQ cutoff scores, we aimed to test the discriminant validity of the HTQ to diagnose cases with BPD symptoms. The PDQ dimensional scores along with the depression and interpersonal sensitivity subscales of the Symptom Checklist-90-Revised Form (SCL-90-R), the Personality Inventory for DSM-5- Brief Form (PID-5-BF), the NEO Five-Factor Inventory (NEO-FFI), and the Emotion Regulation Questionnaire (ERQ) were used to test the criterion validity of the HTQ. Because of the high overlap of hyperbolic temperament with depression, interpersonal sensitivity, negative affectivity, neuroticism, and emotional dysregulation [1–10], we used the aforementioned self-report instruments. All participants informed consent to participate in the study and received assurances from the research team for confidentiality and data protection. This study follows the declaration of Helsinki and received the code of ethics from the Mind GPS Institute of Kermanshah (ID: MGPSI.EA.IR.1401.1).

### Data measurement

#### Hyperbolic temperament questionnaire (HTQ)

This 11-item scale was designed by Zanarini based on 48 identified features associated with hyperbolic temperament and its interpersonal mediators. The items are rated on a 1–9 Likert scale (ranging from strongly disagree to strongly agree). We focused on the 11 items measuring the core trait of hyperbolic temperament [10]. In initial validation research in the U.S. samples, this scale had a unidimensional structure, satisfactory internal consistency, and strong associations with BPD symptoms in both clinical (*r* = .63) and normal (*r* = .53) samples.

*Personality Diagnostic Questionnaire (PDQ-4)*: Bagby and Farvolden (2004) developed a 100-item [99 items for the Persian version] self-report questionnaire to assess and diagnose symptoms of personality disorders. PDQ-4 is a dimensional scale that evaluates the symptoms of twelve personality disorders including schizotypal (9 items), paranoid (7 items), schizoid (7 items), borderline (9 items), antisocial (7 items), histrionic (8 items), narcissistic (9 items), obsessive-compulsive (8 items), avoidant (7 items), dependent (8 items), depressive (7 items), and negativistic (7 items). The cut-off points for these 12 PDs are 4, 4, 5, 3, 5, 5, 5, 4, 5, 4, 5, and 4, respectively. All questions that receive a positive answer are given a score of 1 and the total score is between 0 and 100 [13]. The cut points of the overall score of the PDQ are as follows: a score of 20 or less for normal samples, a score higher than 20 and less than 50 for patients without significant personality disturbance, and a score of 50 and higher for patients with significant personality disorder. The validity and reliability of this scale have been reported as acceptable in Iranian populations [14]. In the present study, Cronbach’s alpha for the total scale was 0.92, ranging from 0.41 to 0.67 for all subscales. More details for reliability statistics of the PDQ-4 and other research tools can be found in Appendix A.

*Symptom Checklist-90-Revised Form (SCL-90-R)*: This is a 90-item scale developed by Derogatis et al. (1976, 2010) to assess psychological symptoms. The SCL-90-R includes nine subscales: somatization (12 items), obsessive-compulsive disorder (10 items), anxiety (10 items), hostility (6 items), phobic anxiety (7 items), paranoid ideation (6 items), psychoticism (10 items), depression (13 items), and interpersonal sensitivity (9 items). This scale also contains six additional questions that do not belong to any of the subscales. The score of each item is rated on a five-point Likert scale from 0 to 4 (ranging from no discomfort to very severe discomfort) [15, 16]. Previous studies in Iran have shown that the SCL-90-R has good validity and reliability in Persian language samples [17, 18]. In this study, we used only the subscales of depression (α = 0.92) and interpersonal sensitivity (α = 0.87).

#### Personality inventory for DSM-5-Brief form (PID-5-BF)

Krueger et al. (2013) developed a dimensional self-report inventory to assess personality pathology according to DSM-5 Section-III (i.e., Criterion B of the Alternative Model for Personality Disorders). Long and brief forms of the PID-5 consist of 220 items and 25 items, respectively. The brief form includes five subscales of negative affectivity (items 8, 9, 10, 11, 15), detachment (items 4, 13,14, 16, 18), antagonism (items 17, 19, 20, 22, 25), disinhibition (items 1, 2, 3, 5, 6), and psychoticism (items 7, 12, 21, 23, 24). All items are scored directly and each item is given a score between 0 and 3 (ranging from often false to often true) [19]. Validation of long and brief forms of this scale in Iranian populations has been reported previously [20, 21]. In the present study, Cronbach’s alpha for the total scale was 0.89, ranging from 0.66 to 0.76 for all subscales.

#### NEO five-factor inventory (NEO-FFI)

The NEO-FFI is a 60-item self-report questionnaire adapted from the NEO-Personality Inventory (240 items). The questionnaire was developed by Costa & McCrae (1989) to assess five basic personality factors including neuroticism, extraversion, openness, agreeableness, and conscientiousness. Each subscale is evaluated using 12 items and 24 items are scored in reverse. The instrument uses a five-point Likert response format from strongly disagree to strongly agree (score 0 to 4) [22]. Previous reports have found the reliability and validity of this questionnaire acceptable [23, 24]. This questionnaire has good validity among Persian language samples [25]. In the present study, Cronbach’s alpha for the total scale was 0.71, ranging from 0.32 to 0.83 for all subscales.

#### Emotion regulation questionnaire (ERQ)

This 10-item self-report scale was designed by Gross & John (2003) to measure the tendency to regulate emotions. Two subscales of the ERQ include cognitive reappraisal (items 1, 3, 5, 7, 8, 10) and expressive suppression (items 2, 4, 6, 9). The score of each item is determined on a seven-point Likert scale from 1 to 7 (ranging from strongly disagree to strongly agree) [26]. The acceptable validity of this scale in Iranian populations has already been confirmed and reported [27]. In the present study, Cronbach’s alpha for the total scale was 0.81, ranging from 0.75 to 0.78 for both subscales.

### Data analysis

Before performing parametric statistical methods, the non-violation of statistical assumptions was checked. Skewness and kurtosis, which were between −1 and + 1, addressed the normality of the data (Appendix A). Analyses were performed in several steps to confirm unidimensionality, calculate reliability, and examine criterion validity. Regarding the first objective, we used an exploratory factor analysis (EFA) and confirmatory factor analysis (CFA) to determine whether a single latent factor could account for covariance among the items. We used the Hull method and Minimum Average Partial (MAP) with maximum likelihood (ML), Unweighted Least Squares (ULS), and Minimum Rank Factor Analysis (MRFA) estimation methods items with oblique rotations in the NPD sample to identify the number of factors [28]. We estimated the number of latent factors across the estimation models by eigenvalues equal to and greater than one. A goodness fit index (GFI) equal to or higher than 0.90, a root mean square error of approximation (RMSEA) smaller than 0.80, a non-normed fit index (NNFI) equal to or higher than 0.90, and a significant chi-square value at the level of less than 0.05 were considered to extract an acceptable structure. We also compared the obtained factor loadings with the results of the initial validation study [10].

The second objective was to test the reliability of the items and the total scale. Regarding this research objective, the reliability of the items in all three samples and the total sample was estimated with Cronbach’s alpha. We considered an alpha value of 0.91 and above as strong and an alpha value of 0.71 to 0.90 as good [29].

Regarding the third objective, we used analysis of variance (ANOVA) and post hoc Tukey test to compare HTQ scores across the samples (discriminant validity) and computed Pearson correlations between the HTQ and validating measures (criterion validity) in the total sample. Finally, linear regression techniques were used to examine the specificity of the HTQ and other potentially overlapping clinical measures to borderline phenomenology in the full sample. We reported the *R*^2^ and Beta statics for all regression analyses. Except for factor analysis, all analyses were performed using the SPSS-20 software. Because the SPSS does not provide CFA indicators, we used the tenth version of the FACTOR software [30] for all factor analyses. All tests were two-tailed and effects at *p* ≤ .05 were interpreted as statistically significant.

## Results

### Construct validity

A single-factor structure with an eigenvalue > 1 was extracted, which accounted for 20 to 39% of the variance and indicated an acceptable fit (GFI > 0.90; RMSEA < 0.07; Chi-square *p-*value < 0.05) across all estimation models in all samples. However, the NNFI was slightly lower than the acceptable level (i.e., around 0.80 across all estimation models). Pattern coefficients for this model in the combined NPD and BPD samples are reported in Table 1, alongside those reported in the initial validation study [10]. As can be seen in Table 1, all items had strong loadings on the higher-order factor, and the pattern coefficients were very similar to those reported by Hopwood et al. [10].

**Table 1:** Pattern coefficients for HTQ items in Persian NPD and BPD samples and original validation study in U.S. student and patient samples

HTQ Items	Present study		Previous study	
	NPD (n = 194)	BPD (n = 104)	Students (n = 545)	Patients (n = 316)
1. I get upset very easily.	0.62	0.65	0.78	0.75
2. I often make a big deal out of things.	0.62	0.69	0.62	0.63
3. I cannot forget my pain or problems.	0.51	0.72	0.57	0.40
4. I have a great deal of trouble letting things go.	0.62	0.62	0.59	0.45
5. I frequently feel that people are insensitive to my feelings.	0.60	0.71	0.50	0.35
6. I am deeply attached to my past and all its painful memories.	0.57	0.68	0.69	0.56
7. My feelings are very easily hurt.	0.68	0.77	0.60	0.63
8. I am a very sensitive person.	0.64	0.62	0.45	0.50
9. I am a nervous or anxious person.	0.64	0.64	0.69	0.60
10. I am a fretful person.	0.66	0.29	0.52	0.42
11. I am often fearful or frightened.	0.63	0.49	0.61	0.54

Abbreviations. HTQ: Hyperbolic Temperament Questionnaire, NPD: Non-Personality Disorder Sample, BPD: Borderline Personality Disorder Sample

### Reliability

Table 2 shows item characteristics across each of the three sub-samples and the total sample. The average score of the items ranges from 3.5 to 4.9. The total scale had acceptable internal consistency values across the samples (*α* = 0.87 to 0.91). The results of this table also show Cronbach’s alpha if the item is deleted. In total, Cronbach’s alpha for all items equals 0.90 or higher.

**Table 2:** Item characteristics across samples

HTQ items	Mean (SD)	Skewness	Kurtosis	Cronbach’s alpha if the item deleted			
				NPD (n = 194)	BPD (n = 104)	OPD (n = 260)	Total (n = 558)
1	4.62 (2.24)	−0.04	−1.00	0.856	0.867	0.875	0.906
2	4.19 (2.12)	0.16	−0.92	0.856	0.862	0.871	0.904
3	4.81 (2.26)	0.03	−0.94	0.865	0.861	0.872	0.905
4	4.27 (2.10)	0.16	−0.83	0.857	0.865	0.867	0.901
5	4.18 (2.11)	0.31	−0.58	0.859	0.861	0.875	0.903
6	4.22 (2.25)	0.26	−0.90	0.860	0.864	0.869	0.901
7	4.79 (2.13)	0.01	−0.73	0.852	0.858	0.870	0.901
8	4.89 (2.30)	0.01	−0.90	0.856	0.869	0.869	0.903
9	4.51 (2.30)	0.08	−1.01	0.857	0.862	0.886	0.900
10	3.55 (2.12)	0.56	−0.54	0.857	0.883	0.881	0.907
11	3.70 (2.22)	0.44	−0.83	0.858	0.872	0.875	0.904
Total scale	47.74 (17.59)	0.05	−0.61	0.869	0.877	0.882	0.911

Abbreviations. HTQ: Hyperbolic Temperament Questionnaire, NPD: Non-Personality Disorder Sample, BPD: Borderline Personality Disorder Sample, OPD: Other Personality Disorder Sample, SD: Standard Deviation

### Criterion and discriminant validity

Table 3 shows the mean and standard deviation scores of validating measures and correlations with mean differences of the HTQ in the total sample. The HTQ score was positively related to all PID-5 and PDQ-4 subscales except antagonism, schizoid, schizotypal, antisocial, and narcissistic personality disorders (all *p* < .05). The HTQ score was also positively correlated with both NEO-FFI neuroticism and SCL90-R depression and interpersonal sensitivity (all *p* < .001). However, significant negative correlations were seen between HTQ and NEO-FFI extraversion (*p* < .001) and agreeableness (*p* = .026). The HTQ score was not significantly correlated with the ERQ subscales and total score. Table 3 also shows the mean scores of the HTQ across the groups. The ANOVA results indicate that the samples differed in the total score of HTQ, with scores for the BPD group being significantly higher than that of the OPD group, which was in turn significantly higher than that of the NPD group (all *p* < .001).

**Table 3:** Correlations between the HTQ and validating measures in the total sample (n = 558) and HTQ differences across subsamples

Measurement	Total sample		HTQ correlation	
	Mean	*SD*	*r*	*P*
PID Negative Affectivity	6.16	2.72	0.280	< 0.001
PID Detachment	5.30	2.63	0.087	0.039
PID Antagonism	4.32	2.86	0.040	0.345
PID Disinhibition	5.48	2.59	0.125	0.003
PID Psychoticism	5.51	2.81	0.126	0.003
NEO-FFI Neuroticism	23.72	5.62	0.369	< 0.001
NEO-FFI Extraversion	27.50	5.67	− 0.135	< 0.001
NEO-FFI Openness	24.61	4.45	0.003	0.941
NEO-FFI Agreeableness	28.65	5.00	− 0.094	0.026
NEO-FFI Conscientiousness	31.79	7.46	− 0.056	0.184
PDQ Paranoid	2.99	1.78	0.161	0.002
PDQ Schizoid	2.43	1.50	0.025	0.563
PDQ Schizotypal	2.91	1.96	0.049	0.248
PDQ Antisocial	1.78	1.72	0.004	0.917
PDQ Borderline	2.62	2.07	0.142	< 0.001
PDQ Narcissistic	2.81	1.79	0.044	0.299
PDQ Histrionic	2.59	1.62	0.146	< 0.001
PDQ Avoidant	2.03	1.67	0.189	< 0.001
PDQ Dependent	1.85	1.83	0.148	< 0.001
PDQ Obsessive-Compulsive	2.95	1.67	0.110	0.009
PDQ Negativistic	2.30	1.60	0.155	< 0.001
PDQ Depressive	2.48	1.73	0.222	< 0.001
PDQ Total	29.75	14.54	0.164	< 0.001
SCL90-R Depression	17.10	10.95	0.243	< 0.001
SCL90-R Interpersonal Sensitivity	10.76	7.09	0.252	< 0.001
ERQ Cognitive Reappraisal	27.31	6.12	− 0.048	0.257
ERQ Expressive Suppression	14.99	4.88	0.002	0.965
ERQ Total	42.30	9.33	− 0.031	0.472
HTQ*	47.74	17.59		
NPD (n = 194)	35.67	13.47		
BPD (n = 104)	60.49	15.24		
OPD (n = 260)	51.65	15.75		

* Note. Analysis of variance (ANOVA) to compare the mean score of HTQ between samples showed significant differences (*F* = 110.75, BPD > OPD > NPD, all *p* < .001)

Abbreviations. HTQ: Hyperbolic Temperament Questionnaire, NPD: Non-Personality Disorder Sample, BPD: Borderline Personality Disorder Sample, OPD: Other Personality Disorder Sample, PID: Personality Inventory for DSM-5, ERQ: Emotion Regulation Questionnaire, NEO-FFI: Neuroticism-Extraversion-Openness Five-Factor Inventory, PDQ: Personality Diagnostic Questionnaire, SCL90-R: Symptom Checklist-90-Revised Form, SD: Standard Deviation

Table 4 shows the results of four linear regression analyses to predict PDQ borderline symptoms by HTQ and other potentially overlapping clinical measures including NEO-FFI neuroticism, PID-5 negative affectivity, SCL90-R depression, and SCL90-R interpersonal sensitivity in the total sample. The HTQ was strongly related to the PDQ borderline in all four models (*β* = 0.324 to 0.395, all *p* < .001), even when controlling for these other variables.

**Table 4:** Linear regression analyses to predict PDQ borderline symptom severity by HTQ and overlapping clinical measures in the full sample

Variables	*R* ^*2*^	Beta	*p*
PDQ Borderline	0.366		< 0.001
HTQ		0.324	< 0.001
NEO-FFI Neuroticism		0.358	< 0.001
PDQ Borderline	0.357		< 0.001
HTQ		0.366	< 0.001
PID Negative Affectivity		0.322	< 0.001
PDQ Borderline	0.360		< 0.001
HTQ		0.389	< 0.001
SCL90-R Depression		0.316	< 0.001
PDQ Borderline	0.349		< 0.001
HTQ		0.395	< 0.001
SCL90-R Interpersonal Sensitivity		0.295	< 0.001

Abbreviations. HTQ: Hyperbolic Temperament Questionnaire, PDQ: Personality Diagnostic Questionnaire, SCL90-R: Symptom Checklist-90-Revised Form

## Discussion

The hyperbolic temperament model, which is characterized as a tendency to experience intense inner pain in response to perceived interpersonal disappointment or frustration, was an attempt to integrate the emotion dysregulation and interpersonal hypersensitivity models [1, 2, 31]. The conceptualization of hyperbolic temperament is strongly linked to often dramatic and impulsive behaviors as well as chronic dysphoria in patients with borderline personality disorder [5, 6, 31]. In the first step, Hopwood et al. [10] formally designed and standardized the HTQ among clinical and non-clinical samples. However, we did not find any other study that validated the HTQ in other cultures. The present study was conducted to investigate the reliability and validity of the 11-item version of the Hyperbolic Temperament Questionnaire (HTQ) in an Iranian sample. Factor analyses replicated the finding that a single factor explains most of the reliable covariance of HTQ items. Moreover, item loadings showed a strong similarity between results previously obtained in U.S. non-clinical and clinical samples [10], and the scale had high levels of internal consistency across three sub-samples. This result supports the interpretation of hyperbolic temperament as a unitary trait involving the tendency to experience and seek validation of negative emotions. The high similarity of item loadings in the present sample with the U.S. samples can indicate the universality of hyperbolic temperament and its measurement tool. However, there are some differences. For example, in the initial validation study [10], item 1 (i.e., *“I get upset very easily”*) showed the strongest factor loading on the general factor, while in the present sample, item 7 (i.e., *“my feelings are very easily hurt”*) loaded more strongly on the general factor. Although temperament models mainly emphasize the role of heredity in the formation of adaptive or maladaptive predispositions [32], hyperbolic temperament is largely influenced by kindling events. Therefore, the role of cultural differences and environmental context in the formation and changes of hyperbolic temperament are more important. Future research in other cultures will address the universality or uniqueness of hyperbolic temperament and it will help the generalizability of the results of the present study and the initial validation study.

People with elevated scores on a screening measure of BPD had the highest HTQ scores, followed by people with elevated scores on screeners of other personality disorders, followed by people without such elevations. This finding supports the validity of the HTQ for identifying people at risk for personality disorder, and in particular the specificity of HTQ to individuals at risk for BPD diagnoses. This finding also further supports the similarity of the HTQ in Iranian samples. The unidimensional nature of HTQ highlights the role of some less-studied underlying mechanisms of personality disorders such as hyperbolic temperament. Although HTQ was originally conceptualized for borderline psychopathology, the links between hyperbolic temperament and symptoms of other personality disorders provide clinicians with a broader theoretical understanding. The relatively similar factor pattern of HTQ in U.S. and Iranian samples addresses the potential role of the unidimensional structure of hyperbolic temperament in the development and persistence of personality psychopathology.

We conducted additional analyses to examine the criterion validity of the extracted factor (i.e., hyperbolic temperament) with other validating measures including the PID-5, PDQ-4, NEO-FFI, ERQ, and SCL90-R subscales of depression and interpersonal sensitivity. The present findings showed that HTQ is positively correlated with the PID-5 subscales of negative affectivity, disinhibition, psychoticism, and detachment. As expected, the negative affectivity strongly and disinhibition, somewhat milder, were psychopathological domains related to the HTQ. Because these two domains are strongly linked with borderline features in the Hierarchical Taxonomy of Psychopathology (HiTOP) [33, 34], borderline features can address some pathways between them and hyperbolic temperament. The correlation between neuroticism or emotional instability as a key component of BPD [5, 8, 9] and HTQ points to an indirect link between borderline features and hyperbolic temperament. These results and previous reports [5, 6, 10] are also consistent with the existence of a strong correlation between HTQ and neuroticism. We found that HTQ is positively correlated with both symptoms of several personality disorders and clinical symptomatology including depression and interpersonal sensitivity. These findings, which are consistent with the previous reports [5, 10, 35], may support a potential link between hyperbolic temperament and a psychopathology general factor. However, the significant strong relationships between hyperbolic temperament and other psychopathological constructs may affect the diagnostic and therapeutic processes of patients with BPD. For example, traditional and modern diagnostic systems are respectively focused on phenotypic symptoms and transdiagnostic constructs such as negative affectivity. Despite the high overlap between negative affectivity and hyperbolic temperament, there are some important differences between these constructs [5]. Failure to recognize the difference between these structures by clinicians can prevent satisfactory treatment results.

The extensive associations obtained from the present study support the convergent validity of the HTQ. Consistent with the research literature [6, 10, 35], we found that hyperbolic temperament was positively related to most maladaptive measures of personality and psychopathology, such as neuroticism, negative affectivity, depression, and interpersonal sensitivity. These findings were expected due to the maladaptive nature of hyperbolic temperament. Also, the negative correlations of the adaptive traits of extraversion and agreeableness with HTQ indicate an acceptable divergent validity. Again, the negative relationship between hyperbolic temperament and adaptive personality constructs was expected for us because the maladaptive temperament has a high overlap with interpersonal sensitivity, which is the opposite of agreeableness. This finding is consistent with previous studies that reported a negative relationship between hyperbolic temperament and extraversion and agreeableness [10, 35].

Our results indicated that the samples differed in the total score of HTQ, with scores for the BPD group being significantly higher than that of the OPD group, which was in turn significantly higher than that of the NPD group. This finding confirms the discriminant validity of HTQ in distinguishing Iranian samples with borderline psychopathology from those with other personality disorder symptoms or low levels of personality disorder symptoms. We also found that the HTQ incremented neuroticism, negative affectivity, depression, and interpersonal sensitivity in predicting borderline features. This finding suggests the unique role of hyperbolic temperament in borderline psychopathology [5, 10, 36, 37].

This was the first attempt to validate the HTQ in a non-Western sample. In general, results in this sample were very similar to those previously reported in U.S. samples [10]. Thus, this study provides evidence for the use of the measure in Iran. Some of the strengths of the present study are as follows: the rigorous translation process, the use of multiple validating measures, and the comprehensive analysis methods. However, there are some limitations. The most important limitation of the present study was not including clinical patients with an established diagnosis. We used a validated screening measure to identify individuals at risk for certain personality disorders, but clinical cases with an established personality disorder diagnosis and the use of structured diagnostic interviews would be a more appropriate benchmark. Meanwhile, the participants were selected by a non-random sampling method. However, using random sampling methods, especially for non-clinical samples, can facilitate the generalizability of the results. The generalizability of the results can also be affected by the target cities for selecting samples because Kermanshah province includes 14 cities and the data was collected only from four cities. Another limitation is the cross-sectional design and our use of self-report instruments for data collection. Self-report measures may be prone to certain systematic biases, and multi-method assessment would help further establish the validity of the HTQ. A multi-assessment approach can include both self-report scores by standardized questionnaires and structured clinical interviews conducted by two or more clinicians to improve diagnostic reliability. A longitudinal study could evaluate the test-retest reliability and longitudinal correlates of HTQ scores and thus the role of hyperbolic temperament in the development of BPD diagnosis in more detail. A recent meta-analysis suggests that the stability of BPD diagnosis over time is about 45% [38]. Studying longitudinal relationships between hyperbolic temperament and BPD symptoms could provide more reliable data to understand any causal relationship. In sum, trying to replicate this study across cultures by considering the current methodological limitations can prevent over-generalization of the findings.

## Conclusion

This study established the reliability and validity of the Hyperbolic Temperament Questionnaire (HTQ) as a measure of the tendency to experience and need validation for negative emotions in an Iranian sample. The 11-item HTQ had a unidimensional structure and showed strong internal consistency. It distinguished individuals at risk for BPD from other samples and had strong correlations with other measures of negative affectivity, personality disorder, emotion regulation difficulties, and interpersonal sensitivity. Because Iran is a country with limited mental health resources and documentation, especially for the prevalence and etiology of personality disorders [39], using this short self-report measure for rapid screening of cases with BPD before common procedures such as clinical interviews helps to save diagnostic and therapeutic time and costs. Future research on the clinical cutoff scores of the HTQ can contribute to the initial screening of patients with BPD symptoms in Iran. Future efforts to validate the HTQ in other non-Western cultures could develop and advance the scientific study of hyperbolic temperament as a mechanism of borderline psychopathology.

### Electronic supplementary material

Below is the link to the electronic supplementary material.

Supplementary Material 1

## Data Availability

The current study data are available on reasonable request to S.K., S_komasi63@yahoo.com.

## References

[1] Zanarini MC, Frankenburg FR (2007). The essential nature of borderline psychopathology. J Pers Disord.

[2] Zanarini MC, Frankenburg FR (1994). Emotional hypochondriasis, hyperbole, and the borderline patient. J Psychother Pract Res.

[3] Zanarini MC, Frankenburg FR (1997). Pathways to the development of borderline personality disorder. J Pers Disord.

[4] Elsner D, Broadbear JH, Rao S (2018). What is the clinical significance of chronic emptiness in borderline personality disorder?. Australas Psychiatry.

[5] Yalch MM, Hopwood CJ, Zanarini MC, Choi-Kain L, Gunderson J (2015). Hyperbolic temperament as a distinguishing feature between borderline personality disorder and mood dysregulation. Borderline Personality and Mood Disorders.

[6] Hopwood CJ, Zanarini MC (2012). The contributions of neuroticism and childhood maltreatment to hyperbolic temperament. J Pers Disord.

[7] Hepp J, Lane SP, Carpenter RW, Niedtfeld I, Brown WC, Trull TJ (2017). Interpersonal problems and negative affect in Borderline Personality and Depressive Disorders in daily life. Clin Psychol Sci.

[8] Linehan M (1993). Cognitive–behavioral treatment of borderline personality disorder.

[9] Crowell SE, Beauchaine TP, Linehan MM (2009). A biosocial developmental model of borderline personality: elaborating and extending Linehan’s theory. Psychol Bull.

[10] Hopwood CJ, Thomas KM, Zanarini MC (2012). Hyperbolic temperament and borderline personality disorder. Personal Ment Health.

[11] Bertsch K, Krauch M, Stopfer K, Haeussler K, Herpertz SC, Gamer M (2017). Interpersonal threat sensitivity in borderline personality disorder: an eye-tracking study. J Pers Disord.

[12] Hopwood CJ, Donnellan MB, Zanarini MC (2010). Temperamental and acute symptoms of borderline personality disorder: associations with normal personality traits and dynamic relations over time. Psychol Med.

[13] Bagby RM, Farvolden P. The personality diagnostic Questionnaire-4 (PDQ-4). In: Hilsenroth MJ, Segal DL, editors. Comprehensive handbook of psychological assessment, Vol. 2. Personality assessment. John Wiley & Sons, Inc.; 2004. pp. 122–33.

[14] Yousefi Z, Gol A, Aghamohammadian H, Seyadzadeh I, Valipur M (2021). Investigating the relationship between early maladaptive schemas and antisocial and borderline personality disorder mediated by pathological hostility and negative emotion. Rooyesh.

[15] Derogatis LR, Rickels K, Rock AF (1976). The SCL-90 and the MMPI: a step in the validation of a new self-report scale. Br J Psychiatry.

[16] Derogatis LR, Unger R. Symptom Checklist-90‐Revised. In The Corsini Encyclopedia of Psychology (eds I. B. Weiner and W. E. Craighead). 2010.

[17] Bakhshaie J, Sharifi V, Amini J (2011). Exploratory factor analysis of SCL90-R symptoms relevant to psychosis. Iran J Psychiatry.

[18] Anisi J, Babaei S, Barani M, Mohammadlo H, Ebrahimi F (2016). Determine the psychometric properties by Symptom Checklist-90-Revised (SCL-90-R) among military forces. Ebnesina J Med.

[19] Krueger RF, Derringer J, Markon KE, Watson D, Skodol AE (2012). Initial construction of a maladaptive personality trait model and inventory for DSM-5. Psychol Med.

[20] Hemmati A, Rahmani F, Bach B (2021). The ICD-11 personality disorder trait model fits the kurdish population better than the DSM-5 trait model. Front Psychiatry.

[21] Amini M, Motevalizade S, Dabaghi P, Shiasi Y, Lotfi M (2021). Psychometric properties and factor structure of original, short and brief forms of personality inventory for DSM-5 (PID-5) in an iranian sample of adolescents. J Mazandaran Univ Med Sci.

[22] Costa PT, McCrae RR (1989). The NEO-PI/NEO-FFI manual supplement.

[23] Costa PT, McCrae RR (1992). Revised NEO personality inventory (NEO-PI-R) and NEO five-factor inventory (NEO-FFI) professional manual.

[24] Robins RW, Fraley RC, Roberts BW, Trzesniewski KH (2001). A longitudinal study of personality change in young adulthood. J Pers.

[25] Anisi J, Majdian M, Joshanloo M, Gohari-kamel Z (2012). Validity and reliability of NEO five-factor inventory (NEO-FFI) on university students. Int J Behav Sci.

[26] Gross JJ, John OP (2003). Individual differences in two emotion regulation processes: implications for affect, relationships, and well-being. J Pers Soc Psychol.

[27] Foroughi AA, Parvizifard A, Sadeghi K, Moghadam AP (2021). Psychometric properties of the Persian version of the emotion regulation questionnaire. Trends Psychiatry Psychother.

[28] Lorenzo-Seva U, Timmerman ME, Kiers HA (2011). The hull method for selecting the number of common factors. Multivar Behav Res.

[29] Taber KS (2018). The use of Cronbach’s alpha when developing and reporting research instruments in science education. Res Sci Educ.

[30] Baglin J (2014). Improving your exploratory factor analysis for ordinal data: a demonstration using FACTOR. Pract Assess Res Eval.

[31] D’Agostino A, Rossi Monti M, Starcevic V (2018). Models of borderline personality disorder: recent advances and new perspectives. Curr Opin Psychiatry.

[32] Cloninger CR, Cloninger KM, Zwir I, Keltikangas-Järvinen L (2019). The complex genetics and biology of human temperament: a review of traditional concepts in relation to new molecular findings. Transl Psychiatry.

[33] Kotov R, Krueger RF, Watson D, Achenbach TM, Althoff RR, Bagby RM, Brown TA, Carpenter WT, Caspi A, Clark LA, Eaton NR (2017). The hierarchical taxonomy of psychopathology (HiTOP): a dimensional alternative to traditional nosologies. J Abnorm Psychol.

[34] de Francisco Carvalho L, Pianowski G, Bacciotti J, Reis AM (2018). Assessing borderline personality disorder based on the hierarchical taxonomy of psychopathology (HiTOP): dimensional clinical personality inventory 2–BPD. Arch Psychiatry Psychother.

[35] Hopwood CJ, Good EW (2019). Structure and correlates of interpersonal problems and sensitivities. J Pers.

[36] Beatson JA, Rao S (2013). Depression and borderline personality disorder. Med J Aust.

[37] Rao S, Broadbear J (2019). Borderline personality disorder and depressive disorder. Australas Psychiatry.

[38] d’Huart D, Seker S, Bürgin D, Birkhölzer M, Boonmann C, Schmid M, Schmeck K (2023). The stability of personality disorders and personality disorder criteria: a systematic review and meta-analysis. Clin Psychol Rev.

[39] Komasi S (2021). Status of epidemiological data related to personality disorders in iranian clinical and general populations. Middle East J Rehabil Health Stud.

