# Supplementary material for: Validation of the hyperbolic temperament questionnaire in Iran
Source: BMC Psychol. 2023 Oct 5;11:307. doi: 10.1186/s40359-023-01364-3 (PMC10557250; doi:10.1186/s40359-023-01364-3)
Supplement: Supplementary file 1 — Supplementary Material 1 [file 40359_2023_1364_MOESM1_ESM.doc]

**Appendix A.** The instrument reliability statistics (*N* = 558)

| Psychological scales | *N* of items | *α* | MMI | Skewness | Kurtosis |
| --- | --- | --- | --- | --- | --- |
| HTQ Total scale | 11 | .91 | .49 | .05 | −.61 |
| PID-5 Negative Affectivity | 5 | .68 | .29 | .06 | .06 |
| PID-5 Detachment | 5 | .68 | .30 | .04 | −.10 |
| PID-5 Antagonism | 5 | .76 | .39 | .37 | −.14 |
| PID-5 Disinhibition | 5 | .66 | .28 | .01 | −.11 |
| PID-5 Psychoticism | 5 | .74 | .36 | .05 | −.04 |
| PID-5 Total scale | 25 | .89 | .25 | −.22 | .12 |
| NEO-FFI Neuroticism | 12 | .56 | .10 | .00 | .17 |
| NEO-FFI Extraversion | 12 | .63 | .13 | .18 | .13 |
| NEO-FFI Openness | 12 | .32 | .04 | −.19 | .60 |
| NEO-FFI Agreeableness | 12 | .51 | .08 | .24 | .12 |
| NEO-FFI Conscientiousness | 12 | .83 | .30 | .02 | −.58 |
| NEO-FFI Total scale | 60 | .71 | .04 | −.15 | −.31 |
| PDQ Paranoid | 7 | .56 | .15 | .17 | −.72 |
| PDQ Schizoid | 7 | .41 | .09 | .36 | −.32 |
| PDQ Schizotypal | 9 | .59 | .14 | .38 | −.51 |
| PDQ Antisocial | 8 | .62 | .17 | .82 | −.10 |
| PDQ Borderline | 9 | .65 | .17 | .53 | −.44 |
| PDQ Narcissistic | 9 | .50 | .10 | .33 | −.46 |
| PDQ Histrionic | 8 | .44 | .09 | .37 | −.32 |
| PDQ Avoidant | 7 | .59 | .18 | .67 | −.26 |
| PDQ Dependent | 8 | .67 | .21 | .76 | −.29 |
| PDQ Obsessive-Compulsive | 8 | .45 | .09 | .14 | −.48 |
| PDQ Negativistic | 7 | .51 | .13 | .27 | −.79 |
| PDQ Depressive | 7 | .58 | .17 | .51 | −.40 |
| PDQ Total scale | 99 | .92 | .10 | −.05 | −.54 |
| SCL90-R Depression | 13 | .92 | .48 | .54 | −.35 |
| SCL90-R Interpersonal Sensitivity | 9 | .87 | .44 | .66 | .15 |
| ERQ Cognitive Reappraisal | 6 | .78 | .38 | −.30 | .11 |
| ERQ Expressive Suppression | 4 | .75 | .42 | .08 | −.33 |
| ERQ Total scale | 10 | .81 | .30 | −.08 | .15 |

**Abbreviation_** α: alpha coefficients, ERQ: Emotion Regulation Questionnaire, HTQ: Hyperbolic Temperament Questionnaire, MMI: mean interitem correlations, NEO-FFI: NEO Five-Factor Inventory, PDQ: Personality Diagnostic Questionnaire, PID-5: Personality Inventory for DSM-5, SCL90-R: Symptom Checklist-90-Revised Form, SD: Standard Deviation.
